# Micro‐proteomics with iterative data analysis: Proteome analysis in *C. elegans* at the single worm level

**DOI:** 10.1002/pmic.201500264

**Published:** 2016-01-07

**Authors:** Dalila Bensaddek, Vikram Narayan, Armel Nicolas, Alejandro Brenes Murillo, Anton Gartner, Cynthia J. Kenyon, Angus I. Lamond

**Affiliations:** ^1^ Centre for Gene Regulation and ExpressionSchool of Life SciencesUniversity of DundeeDow StreetDundeeUnited Kingdom; ^2^ Department of Biochemistry and BiophysicsGenentech HallUniversity of CaliforniaSan FranciscoCAUSA

**Keywords:** *Caenorhabditis elegans*, Heat‐shock, Micro‐proteomics, Nematode, Single worm proteomics, Technology

## Abstract

Proteomics studies typically analyze proteins at a population level, using extracts prepared from tens of thousands to millions of cells. The resulting measurements correspond to average values across the cell population and can mask considerable variation in protein expression and function between individual cells or organisms. Here, we report the development of micro‐proteomics for the analysis of *Caenorhabditis elegans*, a eukaryote composed of 959 somatic cells and ∼1500 germ cells, measuring the worm proteome at a single organism level to a depth of ∼3000 proteins. This includes detection of proteins across a wide dynamic range of expression levels (>6 orders of magnitude), including many chromatin‐associated factors involved in chromosome structure and gene regulation. We apply the micro‐proteomics workflow to measure the global proteome response to heat‐shock in individual nematodes. This shows variation between individual animals in the magnitude of proteome response following heat‐shock, including variable induction of heat‐shock proteins. The micro‐proteomics pipeline thus facilitates the investigation of stochastic variation in protein expression between individuals within an isogenic population of *C. elegans*. All data described in this study are available online via the Encyclopedia of Proteome Dynamics (http://www.peptracker.com/epd), an open access, searchable database resource.

## 1 Introduction

Improvements in proteomics technology have increased the depth of proteome coverage, including both the total numbers of proteins and the degree of sequence coverage achieved, leading to reports of near complete proteome measurements 1. Large‐scale proteomics studies on model organisms such as yeast report complete proteome coverage with ∼4000 proteins 2, 3, while studies on mammalian cells report >10 000 proteins 4. The ability to detect large numbers of PTMs has also increased 5, 6, 7, 8, 9.

**Significance of the study**

A major challenge in biology is to understand variation within genetically identical cells and organisms even when environmental conditions are the same. In *C. elegans*, differences in expression of stress response genes at an early stage of life are reported to correlate with longevity, based on analysis of single genes, by quantifying expression using reporter fusions. However, unbiased analysis of such variation at the global proteome level has not been possible, due to technical limitations relating to working with limited sample sizes. In this study, using *C. elegans* as a model system, we describe the development of a streamlined and reproducible ‘micro‐proteomics’ workflow, allowing global proteomics analysis from single worms. This detected and quantified the proteome of a single worm to a depth of ∼3000 proteins covering a dynamic range of protein expression spanning six orders of magnitude. Using statistical approaches, variations in protein expression upon heat‐shock were reliably detected between individual nematodes. Micro‐proteomics will be of value for studying model organisms and for analysing the variation of the proteomes of individual animals in their natural environment.

Proteomic studies routinely analyse extracts derived from hundreds of thousands to millions of cells. Therefore, resulting quantitative proteomics measurements average across a population of cells, masking any variation between individual cells 10 or organisms 11. Ideally, the protein molecules and protein–protein interactions would be detected and quantified in individual cells. However, the dynamic range of protein expression in human cells is estimated to span seven to eight orders of magnitude while the dynamic range covered by a single LC‐MS injection in large‐scale proteomics is currently limited to ∼10^6^ 12. This presents a significant analytical challenge because proteomics does not allow for amplification steps akin to PCR based transcriptomics. While single cell analyses are currently out of the range of proteomics technology, it appeared possible to develop procedures allowing the analysis of samples of less than 5000 cells, which we term ‘micro‐proteomics’. We applied this micro‐proteomics approach to *C. elegans*, an organism where adults comprise ∼2500 somatic and germ cells.

*C. elegans* is an excellent model organism for studying basic biology, thanks to the plethora of genetic reagents, resources, and information that is available. *C. elegans* is also increasingly used for the study of disease phenotypes, as numerous human disease‐related genes have orthologs in worms. Many systematic studies of gene function have been performed in nematodes, including high throughput analyses of RNA expression levels. However, to understand complex biological processes, such as development, disease and aging, direct analysis of the proteome is also required.

With recent advances in MS‐based proteomics, studies on protein‐protein interactions by Co‐IP from *C. elegans* extracts are becoming commonplace 13, 14. Early large‐scale proteome analyses in worms were largely focused on improving genome annotation and provided limited quantitative information about protein abundance 15, 16. More recently, quantitative *C. elegans* proteomic approaches using stable isotope 17, 18, 19 and chemical labeling 20 have been established and used for global proteomics studies on biological responses. These studies can leverage the wide array of existing information and resources available to the *C. elegans* community to direct follow‐up studies based on protein‐based discoveries.

In this study, we report a workflow for micro‐proteomics analyses in *C. elegans*, identifying ∼3000 proteins from single adult worms and quantifying their expression levels across a dynamic range of ∼6 orders of magnitude, detecting inter‐individual fluctuations of protein expression levels.

## 2 Materials and methods

### 2.1 *C. elegans* strains and maintenance

N2 Bristol was used as the wild‐type strain. Worms were maintained at 20°C on nematode growth medium (NGM) plates seeded with *E. coli* strain OP50.

### 2.2 Heat‐shock of *C. elegans*

For heat‐shock assays, ∼25 mid‐L4 animals (“Christmas tree” stage) 21 were picked on to an NGM plate seeded with OP50, after which the plate was incubated at 30ºC for 6 h. Control L4 animals were incubated at 20ºC for the same duration. Biological (≥10) and technical repeats (≥3) were performed. Individual heat‐shocked (or control) animals were then transferred to microfuge tubes containing lysis buffer (see below) and processed as described below.

### 2.3 Protein extraction and proteolytic digestion in solution

For protein extraction, single nematodes were picked and placed immediately into microfuge tubes containing 50 μL of lysis buffer (8 M urea in 100 mM triethyl ammonium bicarbonate (TEAB) pH 8.5, unless otherwise indicated) and flash frozen using liquid nitrogen. They were then thawed, centrifuged briefly, ultrasonicated using a BioRuptor (30 cycles: 30 sec on, 30 sec off), reduced using tris‐carboxyethylphosphine TCEP (25 mM) for 30 min at room temperature, then alkylated in the dark for 30 min using iodoacetamide (50 mM). Total protein was quantified using the EZQ assay (Life Technologies). The lysates were diluted with 100 mM TEAB fourfold for the first digestion with endoprotease Lys‐C, then further diluted 2.5‐fold before a second digestion with trypsin. Lys‐C and trypsin were used at an enzyme to substrate ratio of 1:50 (w/w). The digestions were carried out overnight at 37ºC, then stopped by acidification with trifluoroacetic acid (TFA) to a final concentration of 1% (v:v). Peptides were desalted using C18 Sep‐Pak cartridges (Waters) following manufacturer's instructions, dried and redissolved in 5% formic acid (FA).

### 2.4 Online reverse‐phase liquid chromatography–MS analysis

RP‐LC was performed using a Dionex RSLC nano HPLC (Thermo Scientific). Peptides were injected onto a 75 μm × 2 cm PepMap‐C18 pre‐column and resolved on a 75 μm × 50 cm RP‐ C18 EASY‐Spray temperature controlled integrated column‐emitter (Thermo) using a 4 h multistep gradient from 5 B to 35% B with a constant flow of 200 nL/min as described previously 12. The mobile phases were: 2% ACN incorporating 0.1% FA (Solvent A) and 80% ACN incorporating 0.1% FA (Solvent B). The spray was initiated by applying 2.5 kV to the EASY‐Spray emitter and the data were acquired on a Q‐Exactive Orbitrap (Thermo Scientific) under the control of Xcalibur software in a data dependent mode selecting the 15 most intense ions for HCD‐MS/MS. Detailed description of the data acquisition parameters is provided in supplementary material.

### 2.5 Data analysis

The raw MS data were processed using MaxQuant (version 1.3.0.5). Proteins and peptides were identified using the UniProt *C. elegans* reference proteome database (August 2013) and the *E. coli* database, using the Andromeda search engine 22, 23 with standard search parameters 12. The false discovery rate was set to 1% for positive identification of proteins and peptides.

Data analyses, including iBAQ calculations, were performed using R version 3.1.3 24 employing Rstudio 0.98.1091 and the ggplot2 package for generating graphs 25. Prior to iBAQ calculation, intensity values for each worm were divided by their sums to correct for potential losses during sample preparation. The number of tryptic peptides used to calculate iBAQ values included peptides generated by missed cleavages. For protein groups containing multiple proteins, the iBAQ value presented is the mean of individual proteins. For the heatmap, log_10_‐transformed, normalized iBAQ values were grouped into hierarchical clusters using the “hclust” function from package “stats” for both of the dimensions (proteins and worms). The heatmap was generated using the heatmap.2 function from package “gplots” and shows values scaled across rows. For PCA analysis, log_10_‐transformed, normalized iBAQ values scaled across rows (proteins; min = 0, max = 1, ‐Inf = –1) were used as input for function “prcomp” (package “stats”); Q‐mode was used for the PCA to describe differences between subjects (worms), as opposed to the more classical R‐mode which describes differences across variables (proteins). GO‐term enrichment analysis was performed using the DAVID Functional annotation tool 26, 27. The full *C. elegans* proteome supplied by DAVID was used as the background list. The results were then plotted to reduce redundancy using the REVIGO suite 28.

## 3 Results

### 3.1 Optimization of protein extraction and data acquisition from single nematodes

**Figure 1:**
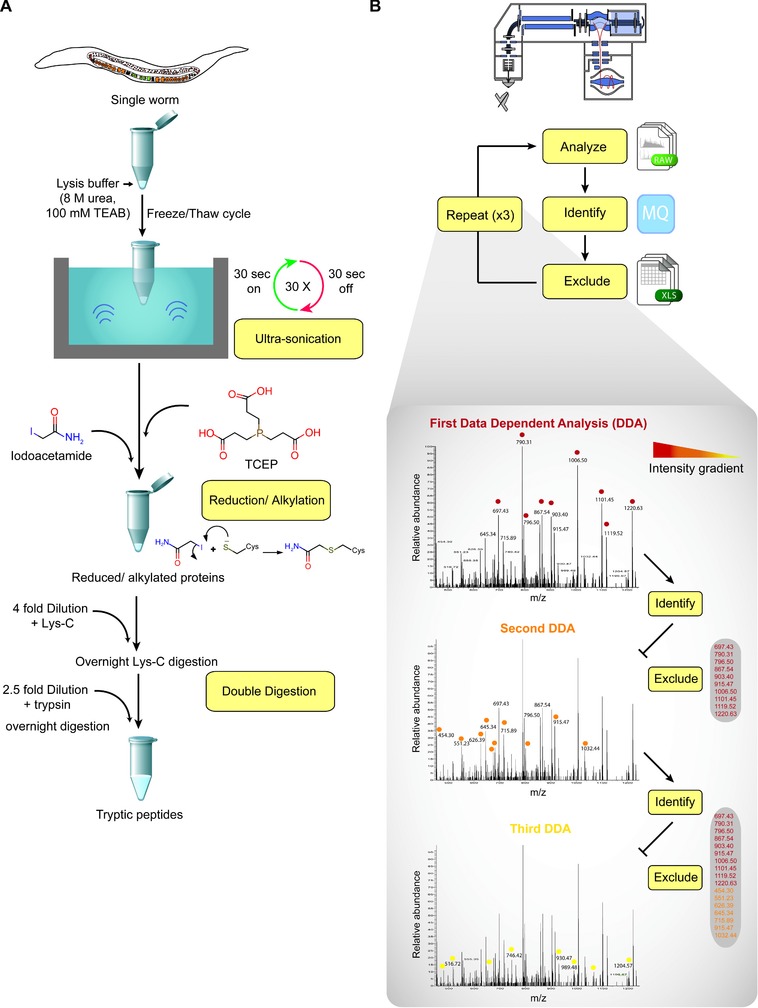
Schematic representation of the micro‐proteomics workflow. (A) Overview of the “one pot” lysis and digestion method. The single worm is placed in a microfuge tube containing lysis buffer and lysed in a sonication water bath. Extracted proteins are reduced and alkylated, then subjected to double digestion using trypsin and lys‐C. (B) Schematic representation of the iterative data acquisition. The proteolytic peptides are divided into three aliquots. The first is analyzed by LC‐MS/MS using standard data‐dependent acquisition and the identified peptides are used to generate an exclusion list. The second aliquot is analyzed using data‐dependent acquisition with the exclusion list generated above. Finally, the third aliquot is analyzed using data dependent acquisition with the concatenated exclusion list from DDA1 and DDA2.

We optimized a lysis workflow that allows proteome extraction from single nematodes, reducing losses using a single reaction pot for lysis, reduction/alkylation and two rounds of proteolytic digestion. The resulting peptide mixture is acidified with TFA and desalted using a Sep‐pak^TM^ cartridge, (Fig. 1A). Individual *C. elegans* animals were picked directly into a fresh microfuge tube containing lysis buffer. Samples were flash frozen in liquid nitrogen and kept at –80ºC at least overnight. The animals were thawed and subjected to 30 cycles of ultrasonication at 4ºC in the same low bind tubes, as described in the Methods section. Lysates were reduced using TCEP for 30 minutes and then alkylated in the dark for 30 min using iodoacetamide (IAA). The lysate was diluted fourfold in the same tube for the first digestion step, then diluted again 2.5‐fold for the second digestion step. Several buffer systems were evaluated before choosing 8 M urea in 100 mM TEAB, pH 8.5 (Supporting Information Fig. 1).

After the single nematode sample preparation workflow was optimized, different acquisition methods were evaluated to maximize the number of peptides sequenced and thus of proteins identified. Single injection and long non‐linear gradients were evaluated (data not shown), along with iterative data‐dependent acquisition (DDA), which increased identification of peptides and proteins (>3000 proteins). The tryptic hydrolysates were redissolved in 50 μL of 5% formic acid (v/v) and divided into three separate injections (summarized in Fig. 1B). The first injection (15 μL) was analyzed using a 4 h gradient and standard DDA method, with the 15 most intense peptides selected for HCD‐MS/MS events. The raw data from the first injection were processed to identify peptides and proteins using MaxQuant 22, 23. Peptides identified in the first step were compiled into an exclusion list and used to avoid sequencing the same peptides in the second data acquisition step analysing a second aliquot. A combined exclusion list was then employed when analysing a third aliquot. This iterative data analysis method improves both the total number and the dynamic range of expression levels of the proteins identified. Due to the random sampling effect in DDA, the total number of peptides identified is increased with additional replicate analysis compared to a single injection. However, there is a further increase in the total number of peptides identified from informed (or iterative) data acquisitions (Supporting Information Fig. 2). While the data demonstrate a clear, albeit small, advantage of using exclusion lists for iterative data analysis, this is currently limited by the size of the exclusion lists that are allowed. We anticipate the benefit of this strategy could increase even more in the future if technical improvements in instrumentation and software allow the construction of more comprehensive exclusion lists.

**Figure 2:**
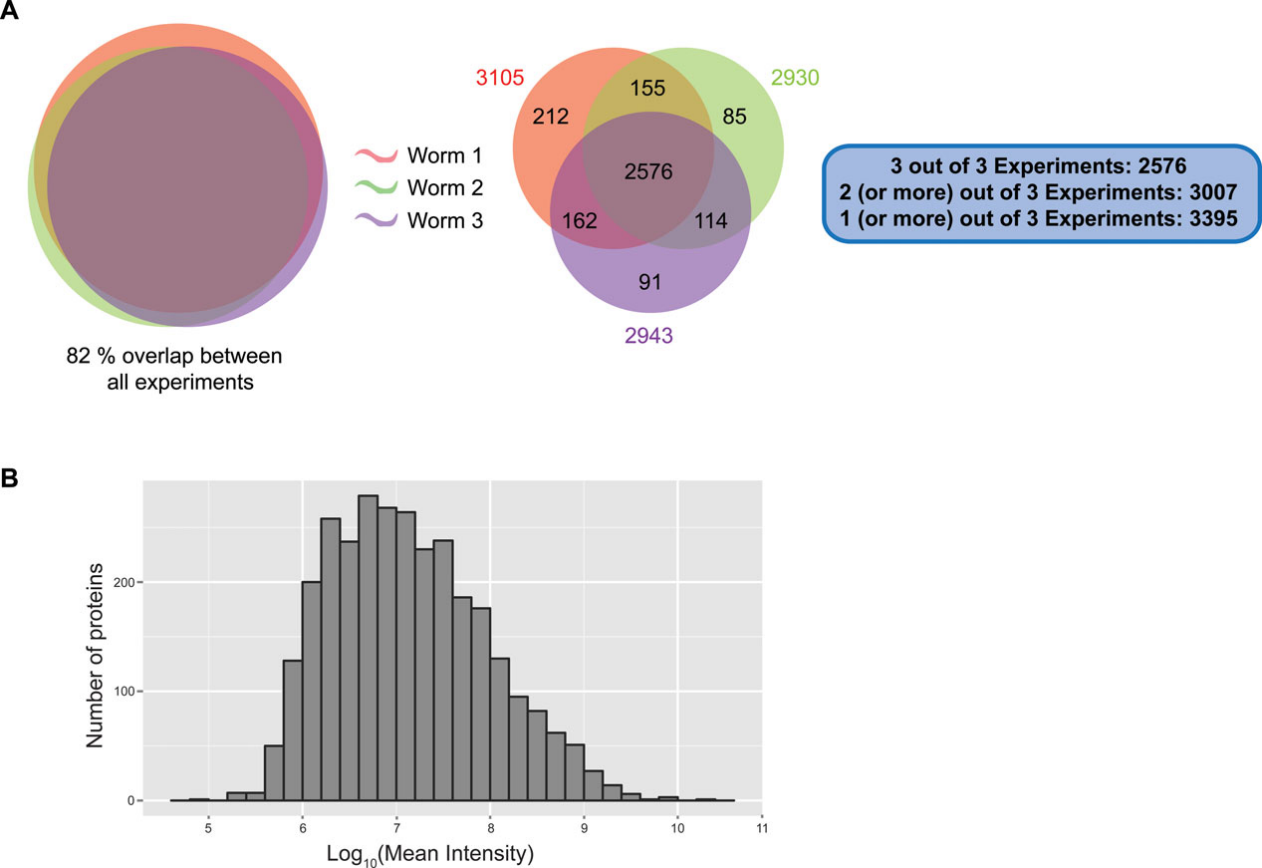
Summary of micro‐proteomics results. (A) Venn diagram comparing the proteins identified from three individual worms using the micro‐proteomics workflow and iterative data acquisition. (B) Distribution of protein intensities in the single worm dataset.

### 3.2 Identification of ∼3000 proteins from a single worm with expression levels spanning six orders of magnitude

By combining the one‐pot sample preparation method (Fig. 1A) and the iterative data acquisition method (Fig. 1B), ∼3000 proteins were reproducibly identified. The protein contents of three single worms, lysed using 8 M urea in 100 mM TEAB and analyzed using the three‐step iterative acquisition method, are compared in a Venn diagram (Fig. 2A). This shows >80% overlap in proteins identified, with protein numbers ranging from 2930 to 3105, covering a dynamic range of expression levels across six orders of magnitude (Fig. 2B).

### 3.3 Chromatin‐associated factors detected by micro‐ and macro‐proteomics

**Figure 3:**
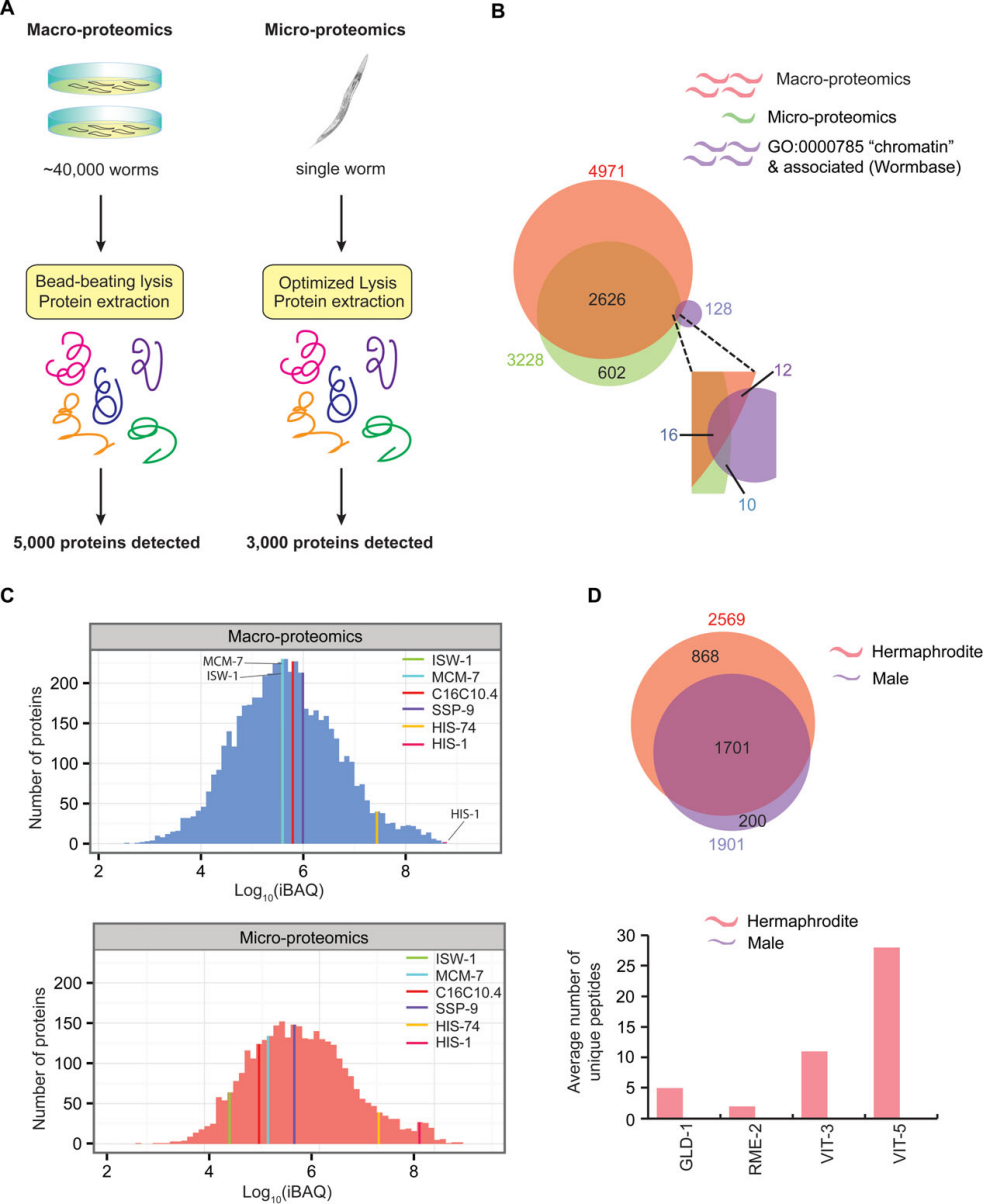
Comparison of micro‐proteomics and macro‐proteomics analysis of *C. elegans*. (A) Comparison of number of worms required and overall number of proteins identified. (B) Venn diagram comparing the total number of proteins and chromatin‐associated proteins identified in the macro‐proteomics and the micro‐proteomics studies. (C) Comparison of the dynamic range of expression levels of proteins identified in each study. (D) Venn diagram comparing the numbers of proteins identified in the hermaphrodite worm and the male worm.

This micro‐proteomic analysis of nematodes identifies >3000 proteins from a single animal, whereas a recent macro‐proteomics study using ∼40 000 pooled nematodes identified ∼5000 proteins (29; Fig. 3A, left panel). Surprisingly, although there is a considerable overlap between proteins identified using macro‐ and micro‐proteomics approaches, 602 proteins were exclusive to the micro‐proteomics dataset (Fig. 3B). This is likely due to differences in sample lysis and preparation; the micro‐proteomics workflow uses a single lysis and digestion buffer, bypassing the need for buffer‐exchange steps that can result in losses.

The micro‐proteomics approach identifies comparable numbers of mid‐high and high abundance nematode proteins to the macro‐proteomics dataset; as expected, the major differences between the two approaches can be attributed to low and low‐mid abundance proteins (Fig. 3C). To compare protein quantification across the datasets, we examined a subset of chromatin‐associated factors that comprise proteins varying in abundance by many orders of magnitude. Label‐free quantification using the iBAQ algorithm 30 showed comparable measurement of highly abundant proteins, including histones HIS‐1 and HIS‐74, using either micro‐, or macro‐proteomics (Fig. 3C). However, there was variability in mid‐ and low‐ abundance proteins. Thus, while some proteins, including SSP‐9 and MCM‐7, showed little variation in levels, others, including ISW‐1 and C16C10.4, differed by up to two orders of magnitude. This may result from inaccurate quantification owing to low sequence coverage of low/mid‐abundance proteins (<10%) in the micro‐proteomics dataset (Supporting Information Fig. 3). However, the differences may also be influenced, at least in part, by the effect of averaging in the macro‐proteomics dataset, particularly if protein levels vary greatly between individuals in the population.

Inspection of *C. elegans* chromatin‐associated factors shows that of the 128 genes annotated by WormBase 31, 32, 33 either with “chromatin”, or associated GO terms (Fig. 3B; Supporting Information Table 1), the protein products of 26 of these genes were detected by micro‐proteomics. This was comparable to the chromatin‐associated proteins detected using macro‐proteomics (28 proteins; Fig. 3B). Thus, many *C. elegans* factors annotated as chromatin‐associated were not detected by either micro‐ or macro‐proteomics approaches, though we note that not all of the cognate genes may be expressed in the adult worms used for these studies.

Next, we compared the proteomes of 3 x day 1 adult hermaphrodites and males. We could quantify 2569 protein groups in the hermaphrodites (two out of three experiments) and 1901 protein groups in the males (Fig. 3D, upper panel). The majority of protein groups were detected in both hermaphrodites and males, with 868 detected in hermaphrodites alone and 200 in males alone. We therefore looked for proteins known to be specific to hermaphrodites, based on data compiled by WormBase (see below), such as vitellogenins, and tested for their absence in males. As expected, vitellogenins including VIT‐3 and VIT‐5, the yolk receptor RME‐2 and the germline‐specific transcriptional repressor essential for oogenesis GLD‐1 34, were detected exclusively in each of the three adult hermaphrodites but not in the males (Fig. 3D, lower panel). To probe for male‐specific genes in our dataset, we mined previous RNA‐based gene expression data from WormBase. Surprisingly, this revealed only a single hit, *lov‐1*, possibly due to poor annotation of adult male‐specific genes. Although the LOV‐1 protein was not detected in the micro‐proteomics dataset, we detected a subset of proteins in the adult male that were not found in the hermaphrodite samples.

In summary, we conclude that the micro‐proteomics approach can distinguish differences between individual nematodes at the level of hermaphrodite‐specific proteins in adult hermaphrodites and their absence in adult males (Fig. 3D).

### 3.4 Variations in the response to heat‐stress amongst individual nematodes

We next studied whether more subtle changes between individuals could be identified. To this end, we compared the effect of heat‐stress on individual worms using 10 x day 1 adult worms for heat‐shock and 10 x day 1 adult worms as a control. The mild heat‐stress used (6 h at 30°C) did not cause appreciable lethality. A previous microscopy study suggested that individual nematodes respond to heat‐stress differently, with some animals showing greater induction of heat‐shock proteins than others. The study also reported that the magnitude of induction of GFP:HSP‐16 after heat‐stress during young adulthood correlated positively with subsequent lifespan 11.

**Figure 4:**
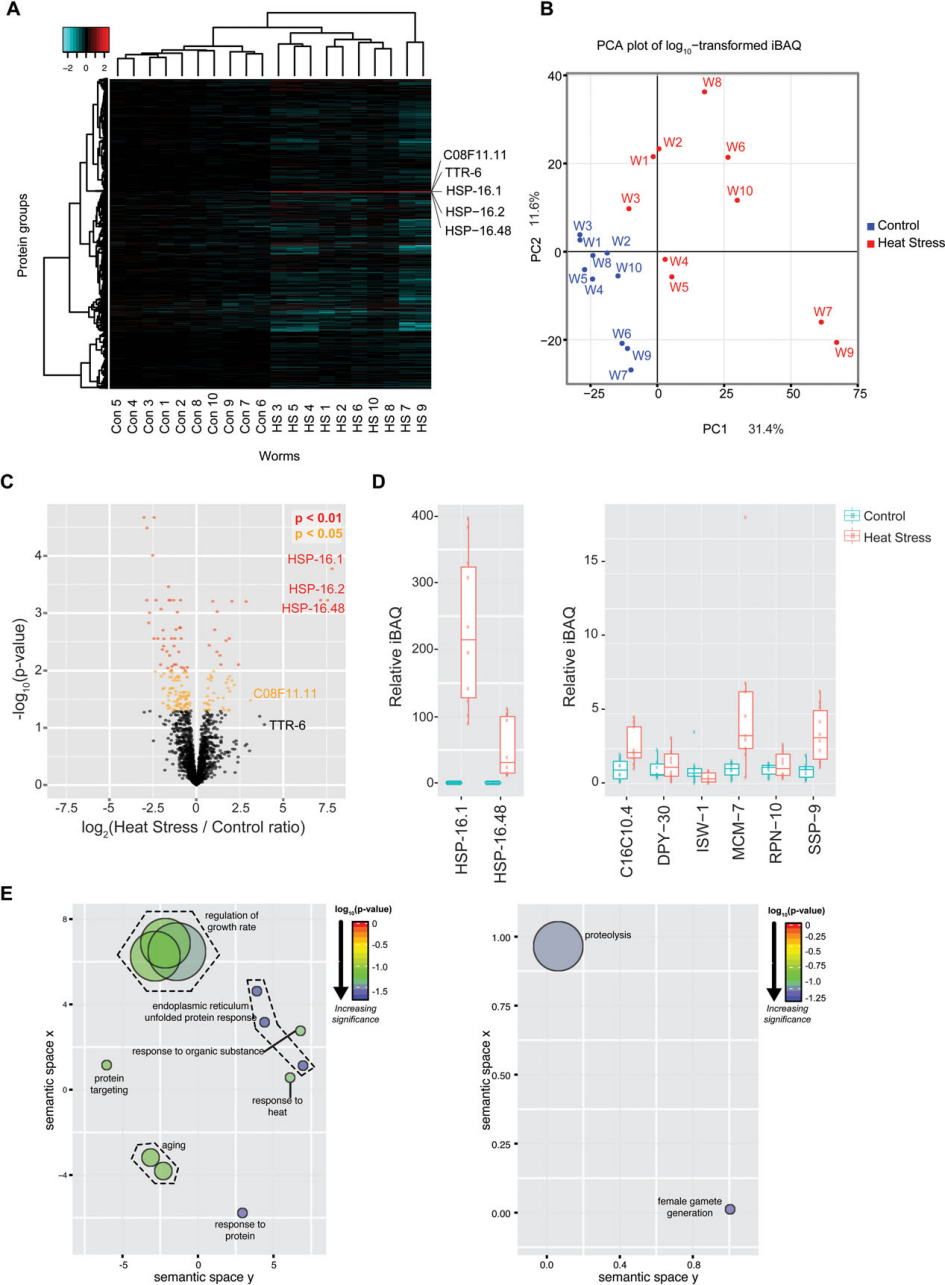
Proteomic analysis of the response of individual worms to heat‐stress. (A) Heatmap of the log_10_ ratio of iBAQ values calculated for proteins identified from ten control (Con) and ten heat‐shocked (HS) worms for each protein group relative to the mean iBAQ value for the ten control worms; only proteins groups detected in all 20 worms are shown. (B) Q‐mode PCA plot based on the log_10_ iBAQ profile of the ten control (Con, blue) and ten heat‐shocked (HS, red) worms (W); only two principal components are represented (43% of total variance). (C) Volcano plot of the log_2_ ratio of mean heat‐stress to control iBAQ values plotted against the negative log_10_ ratio of the *p*‐value calculated using a *t*‐test. The graph is colour coded to indicate the level of significance. (D) Boxplot of the untransformed iBAQ values for ten control (Con) and ten heat‐shocked (HS) worms, relative to the mean value for the ten control worms, for the indicated proteins. (E) Schematic representation of the GO‐term enrichment analysis. GO‐term enrichment analysis was performed using DAVID (ref) for up‐ and down‐regulated proteins (*p*‐value < 0.05; FDR < 1%) after heat‐stress. The DAVID output was further analyzed using ReviGo and plotted using R.

Hierarchical clustering was used to assess differences in response to heat‐shock between the individual nematodes, showing the two populations have distinct protein expression patterns (Fig. 4A). The ten control animals clustered tightly together, distinct from the ten heat‐shocked individuals. Differences in protein levels between individual heat‐shocked worms were much more pronounced than those between control worms. Q‐mode Principal Component Analysis (PCA, Fig. 4B) of the iBAQ intensities measured by micro‐proteomics also shows a more scattered distribution for heat‐shocked worms than control worms for the first two principal components (∼43% of the variance explained). This suggests that in spite of being isogenic and raised in similar environments, individual worms may vary in either their sensitivity, or response, to heat‐shock. In particular, worms 7 and 9 showed a greater response to heat‐shock compared with other treated worms.

Figure 4C shows a volcano plot of mean heat‐stress to mean control iBAQ ratios versus *p*‐value from a *t*‐test for proteins detected in at least six of the ten control and heat‐shocked worms, respectively. Variants of heat‐shock protein 16 (HSP‐16) were among the most robustly induced proteins following heat‐stress, consistent with previous studies (17; Fig. 4C). In keeping with previous studies 11, HSP‐16 family members (here HSP‐16.1, HSP‐16.2 and HSP‐16.48) showed significant variation between individuals following heat‐stress. Some heat‐shocked animals showed ∼fivefold greater induction of HSP‐16.1 compared with others (Fig. 4D, left panel).

Other proteins were strongly up‐regulated upon heat‐shock, including: TTR‐6, a protein known from studies in worms and mice to play a role in the heat‐shock response 20, 35, and C08F11.11, a protein of unknown function(s) that we thus propose may play a role in the heat‐shock response (Fig. 4A). Other proteins also showed expression levels changing significantly in response to heat‐shock, albeit with smaller magnitude (Supporting Information Table 2). The complete list of proteins identified in this study is available in Supporting Information Table 3.

A closer look at specific chromatin‐associated factors (Fig. 3B) reveals proteins, particularly MCM‐7, that are induced after heat‐stress, and, like HSP‐16.1 (Fig. 4D, left panel), that show significant variation amongst individuals in the magnitude of their induction (Fig. 4D, right panel). The abundance of others, including ISW‐1, is relatively unchanged after heat‐stress and does not vary significantly between individuals (Fig. 4D, right panel). Chromatin‐associated protein level changes in response to heat shock are shown in Supporting Information Fig. S4.

GO‐term enrichment of proteins whose abundance changed by >twofold in either direction (with *p*‐values < 0.05 calculated using a *t*‐test corrected for FDR<0.1), confirmed that many of these proteins have previously been associated with heat‐stress. Thus, “endoplasmic reticulum unfolded protein response”, “regulation of growth rate”, “response to protein”, “regulation of growth rate”, “aging” and “response to heat” were among the GO‐terms significantly enriched in the subset of proteins whose abundance increased following heat‐stress (Fig. 4E, left panel).

Two GO‐terms were significantly enriched in proteins whose abundance decreased post heat‐stress (Fig. 4E, right panel). One was “female gamete generation”, consistent with the fact that heat‐stress can reduce *C. elegans’* fertility 36. The other GO term that decreased with heat‐shock was “proteolysis”. This is surprising, given observations that proteins are marked for proteasomal degradation during heat‐stress, resulting in increased protein clearance 37. However, previous reports suggest that degradation via the enzyme proline endopeptidase is decreased during heat‐stress 38. We therefore speculate that non‐proteasomal protein degradation is inhibited during heat‐stress. Indeed, of the five proteins in our dataset annotated with the GO‐term “proteolysis”, four are proteases (metallopeptidases NEP‐22 and NEP‐17, aspartyl protease ASP‐2 and cathepsin B‐like proteinase CPR‐5) and one is involved in protein NEDD8‐conjugation (NEDD8‐activating enzyme E1 catalytic subunit UBA‐3).

In summary, our micro‐proteomics data show variation amongst individual nematodes within an isogenic population in the protein response to heat‐stress. We conclude that micro‐proteomics can be used to characterize the proteomes of individual nematodes to study individual variation within populations.

### 3.5 Data sharing

**Figure 5:**
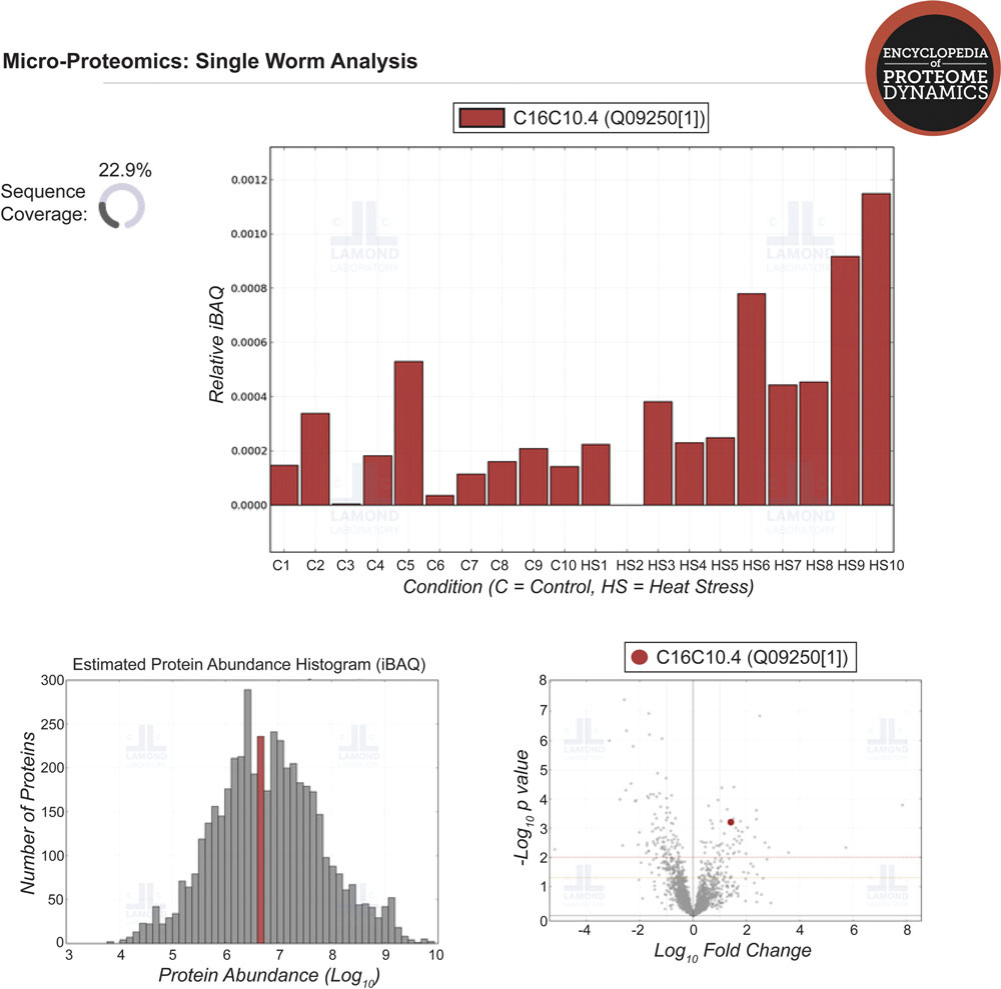
Screenshot showing an example of data visualization available via the Encyclopedia of Protein Dynamics. The EPD (http://www.peptracker.com/epd) is an open access, searchable database of proteomic data.

All of the MS‐based proteomics data described in this study, including the heat‐shock response averaged across ten nematodes, as well as the individual variation between worms, are freely accessible through The Encyclopaedia of Proteome Dynamics (EPD‐http://www.peptracker.com/epd) 39. The EPD provides a searchable online resource for visualising and exploring all the proteomics data with convenient links to WormBase and other related online data repositories. Figure 5 shows a screenshot of the micro‐proteomics data visualization that is possible using the EPD. In addition, the MS data have also been deposited to the ProteomeXchange Consortium 40 via the PRIDE partner repository with the dataset identifier PXD002948.

## 4 Discussion

We have developed a streamlined micro‐proteomics workflow for the analysis of single nematodes that can detect ∼3000 proteins from single worms, spanning a dynamic range of protein expression levels of ∼10^6^. We have used the micro‐proteomics workflow to perform a label‐free quantitative comparison between the proteomes of male and hermaphrodite nematodes. This analysis detected variation in protein expression in distinct groups of worms (i.e., males and hermaphrodites), without such differences being masked by stochastic variation in protein expression between individual worms. We also used micro‐proteomics to compare the heat‐shock response of individual nematodes. PCA analysis on the proteins identified in both control and heat‐treated populations highlighted differences. Furthermore, hierarchical clustering showed that the degree of variation between the control worms was smaller than the variation between control and treated worms, allowing measurement of differences in protein levels upon heat‐stress. Clustering analysis suggests that there may be subsets of response classes following heat‐shock, although establishing this more clearly needs further investigation based on analysis of larger numbers of worms.

The micro‐proteomics workflow minimizes sample handling so that lysis, chemical derivatization and digestion are all carried out in the same reaction pot. This avoids multi‐step lysis and protein precipitation steps with concomitant losses, biases in extraction and possible introduction of contaminants. The micro‐proteomics workflow coincides with the recent trend in simplification and miniaturization of sample preparation, which is an essential step in proteomics analyses of rare and/or sample limited systems 41, 42. For example, by introducing a method using a single reaction tube and magnetic bead‐based sample handling workflow, Hughes et al. recently demonstrated that ∼2000 proteins could be identified, either from a drosophila embryo, or from as little as 1000 HeLa cells 41.

Our data show that variation in biological responses affecting protein expression levels occurs between individuals and that this can now be detected and quantified at a global proteomics level. While a previous microscopy study also reported variation in gene expression between individual worms 11, a proteomics approach on a single organism level is potentially more powerful because it allows thousands of proteins to be analyzed in parallel and detects endogenous proteins without the need for using tags or antibodies.

We anticipate that micro‐proteomics will in future be used to study a variety of regulatory events and biological responses in *C. elegans* and other organisms. When used in combination with conventional worm genetics and microscopy, this provides a powerful strategy for studying regulatory mechanisms and cell phenotypes. Furthermore, we expect the depth of the proteome covered by micro‐proteomics to improve as mass spectrometers become more sensitive and potentially also via future improvements in sample handling, chromatography, data processing and analysis. This can help to open up a new level of proteomic analyses that investigate the role of stochastic variation in protein expression between individuals in a population during response mechanisms.

## Supporting information

As a service to our authors and readers, this journal provides supporting information supplied by the authors. Such materials are peer reviewed and may be re‐organized for online delivery, but are not copy‐edited or typeset. Technical support issues arising from supporting information (other than missing files) should be addressed to the authors.

Figure S1 Venn diagrams comparing the number of proteins identified using different lysis methods in the micro‐proteomics workflow. B) Venn diagrams comparing the number of proteins identified from 50, 10 and 1 worm.

Figure S2 Bar chart comparing informed iterative data acquisition and independent data acquisition on replicates. Peptides identified in the first analysis are shown in white; the number of new peptides identified in the second and third injections are shown in dark grey, and light grey, respectively and are expressed as a percentage of the number of peptides identified in the first injection.

Figure S3 Histograms comparing the distribution of sequence coverage obtained using micro‐proteomics for both the control worms (con) and the heat‐shocked worms (HS), and the distribution of sequence coverage obtained using macro‐proteomics.

Figure S4 Boxplots showing the protein level changes in chromatin associated proteins upon heat‐shock.

Supplementary Table 1. List of chromatin‐associated proteins identified using the micro‐proteomics workflow in single worms.

Supplementary Table 2. List of proteins identified in at least 6 out of 10 worms, with p value cut off of 0.05 and FDR < 0.1.

Supplementary Table 3. Protein Groups identified in the heat shock experiment after MaxQuant analysis.
