# Supplementary figures and images for: Micro‐proteomics with iterative data analysis: Proteome analysis in C. elegans at the single worm level
Source: Proteomics. 2016 Jan 7;16(3):381–92. doi: 10.1002/pmic.201500264 (PMC4819713; doi:10.1002/pmic.201500264)

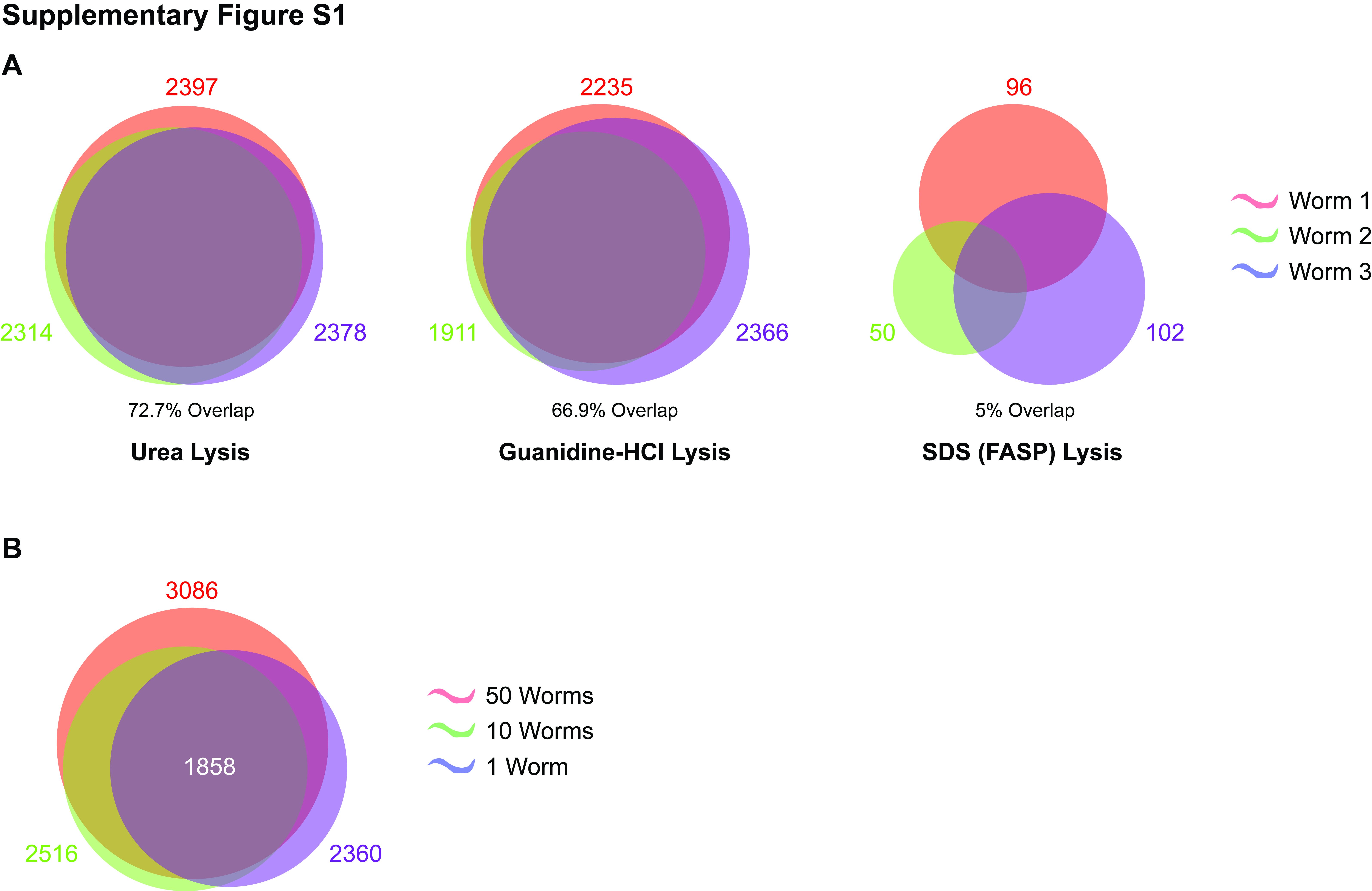

Supplement: Supplementary file 1 — Figure S1 Venn diagrams comparing the number of proteins identified using different lysis methods in the micro‐proteomics workflow. B) Venn diagrams comparing the number of proteins identified from 50, 10 and 1 worm. [file PMIC-16-381-s001.jpg]

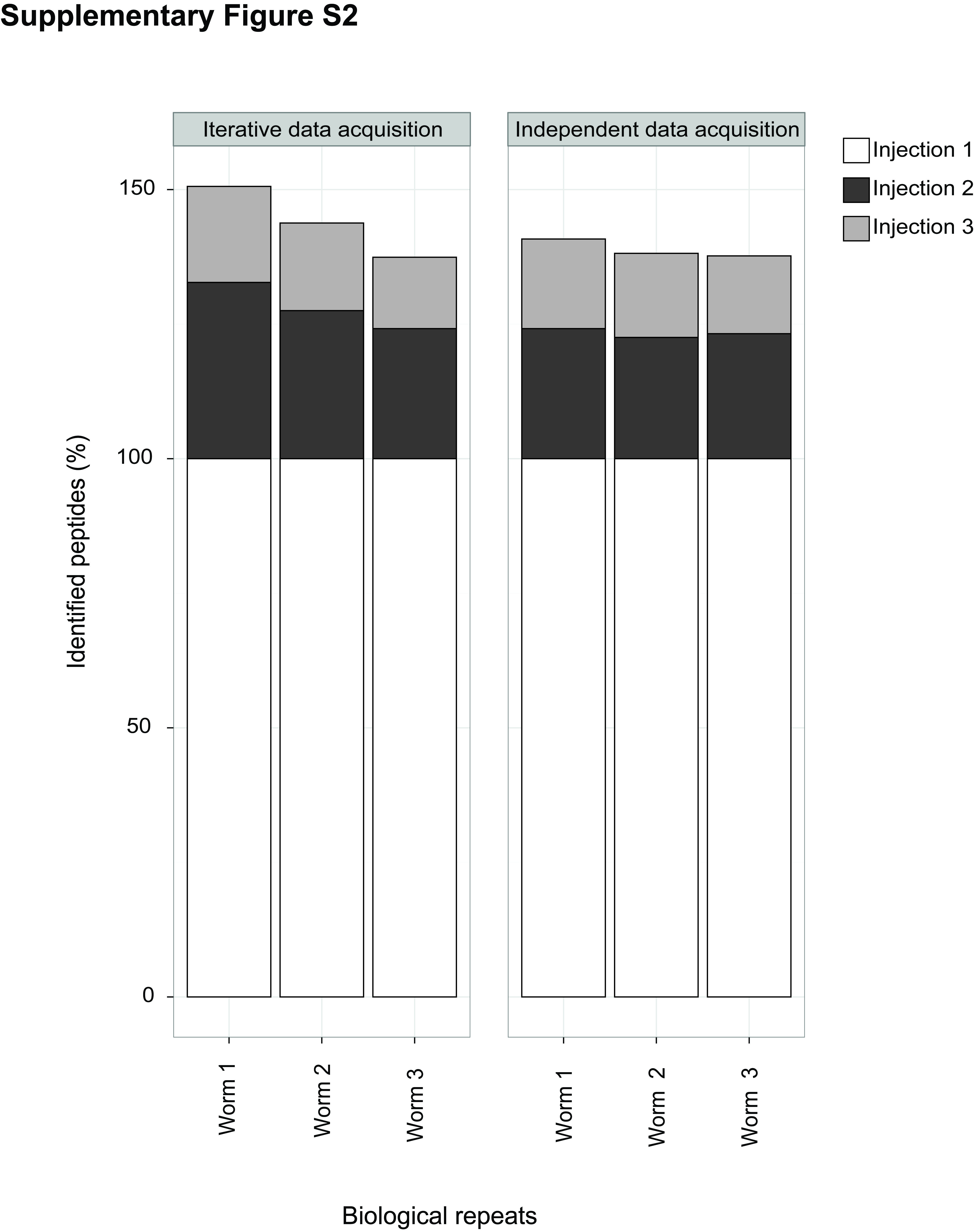

Supplement: Supplementary file 2 — Figure S2 Bar chart comparing informed iterative data acquisition and independent data acquisition on replicates. Peptides identified in the first analysis are shown in white; the number of new peptides identified in the second and third injections are shown in dark grey, and light grey, respectively and are expressed as a percentage of the number of peptides identified in the first injection. [file PMIC-16-381-s002.jpg]

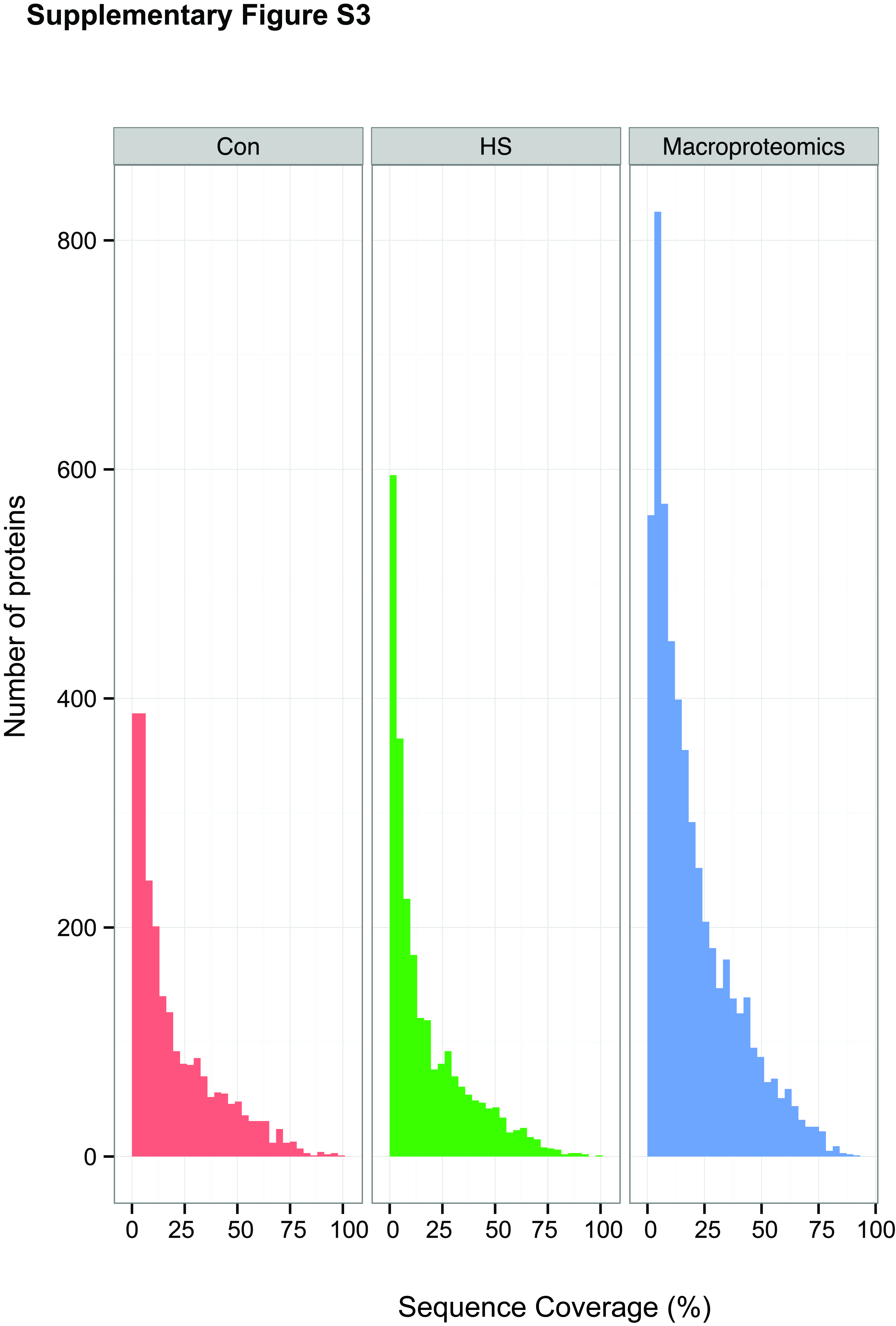

Supplement: Supplementary file 3 — Figure S3 Histograms comparing the distribution of sequence coverage obtained using micro‐proteomics for both the control worms (con) and the heat‐shocked worms (HS), and the distribution of sequence coverage obtained using macro‐proteomics. [file PMIC-16-381-s003.jpg]
