# Supplementary material for: Micro‐proteomics with iterative data analysis: Proteome analysis in C. elegans at the single worm level
Source: Proteomics. 2016 Jan 7;16(3):381–92. doi: 10.1002/pmic.201500264 (PMC4819713; doi:10.1002/pmic.201500264)
Supplement: Supplementary file 4 — Figure S4 Boxplots showing the protein level changes in chromatin associated proteins upon heat‐shock. [file PMIC-16-381-s004.pdf]

PROTEOMICS

Supporting Information for Proteomics

DOI 10.1002/pmic.201500264

Dalila Bensaddek, Vikram Narayan, Armel Nicolas, Alejandro Brenes Murillo, Anton Gartner, Cynthia J. Kenyon and Angus I. Lamond

Micro-proteomics with iterative data analysis: Proteome analysis in *C. elegans* at the single worm level

© 2015 The Authors. *PROTEOMICS* Published by Wiley-VCH Verlag GmbH & Co. KGaA, Weinheim

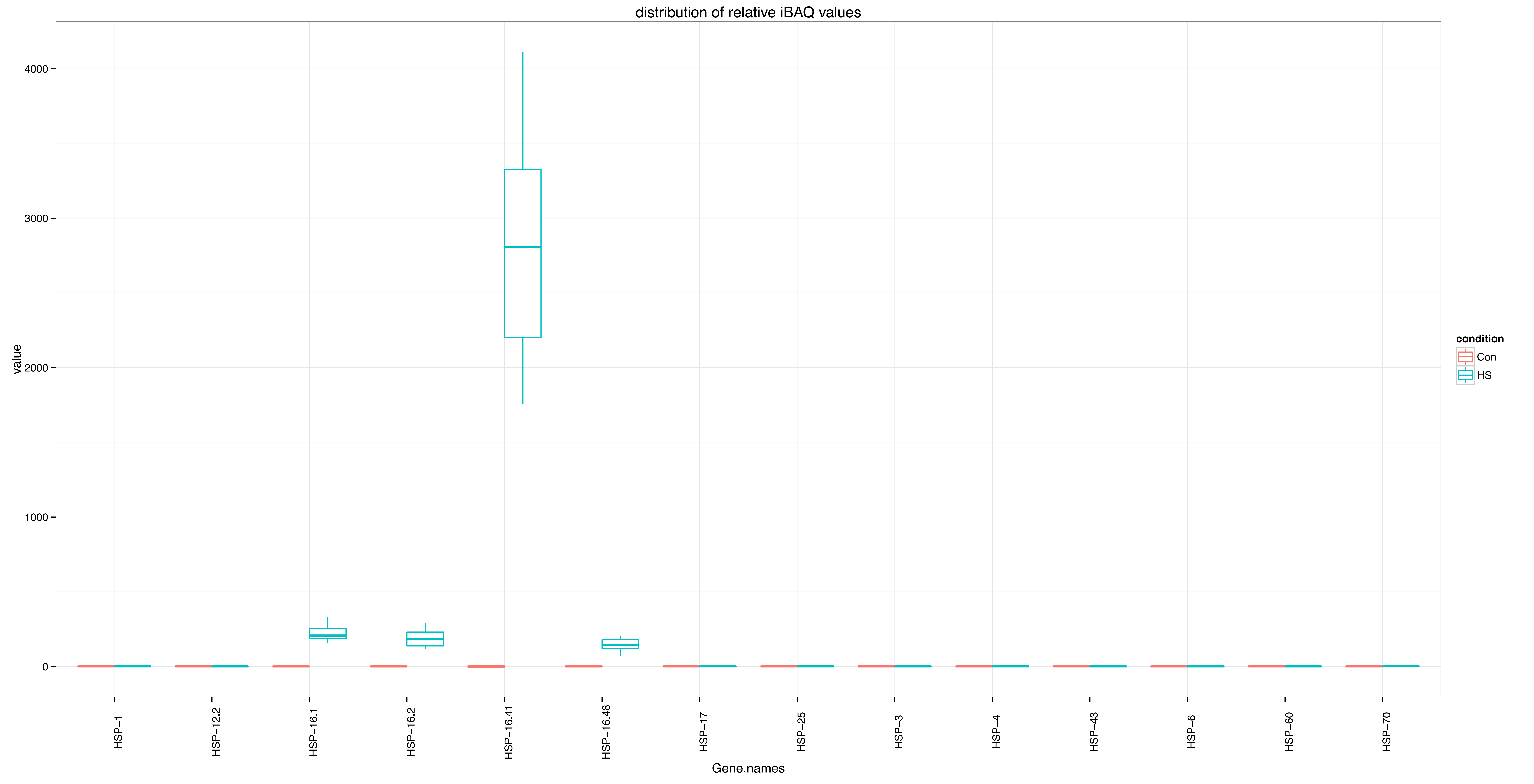

distribution of relative iBAQ values
value
4000
3000
2000
1000
0
Gene.names
HSP−1
HSP−12.2
HSP−16.1
HSP−16.2
HSP−16.41
HSP−16.48
HSP−17
HSP−25
HSP−3
HSP−4
HSP−43
HSP−6
HSP−60
HSP−70
condition
Con
HS
